# Sex-on-premise venues, associated risk behaviors, and attitudes toward venue-based HIV testing among men who have sex with men in Lima, Perú

**DOI:** 10.1186/s12889-020-08604-w

**Published:** 2020-04-19

**Authors:** Alexander Lankowski, Hugo Sánchez, José Hidalgo, Robinson Cabello, Ann Duerr

**Affiliations:** 1 Division of Allergy & Infectious Diseases, Department of Medicine, University of Washington, Seattle, WA USA; 2 Vaccine & Infectious Disease and Public Health Science Divisions, Fred Hutchinson Cancer Research Center, Seattle, WA USA; 3 Epicentro, Lima, Perú; 4 Vía Libre, Lima, Perú; 5 Departments of Epidemiology and Global Health, University of Washington School of Public Health, Seattle, WA USA

**Keywords:** HIV, Sexual behavior, MSM, Perú, Sex-on-premise venue, Sauna, HIV testing, HIV prevention, Sexually transmitted infection

## Abstract

**Background:**

In Perú, HIV disproportionately affects men who have sex with men (MSM). Despite widespread access to treatment, the high rate of new HIV infections has remained unchanged over the last decade. Low knowledge of HIV status associated with late diagnosis is a key factor underlying the high HIV incidence observed in this setting, creating conditions for efficient onward transmission. Improving access to HIV testing and prevention services for those at highest risk is an important public health priority. Sex-on-premise venues (SOPVs) – saunas, sex clubs, pornographic movie theaters, hourly hotels, and bars/discos with areas where sex is permitted – may be opportune sites for outreach; however, further research on SOPVs and the populations who frequent them is needed to inform such efforts.

**Methods:**

We conducted a cross-sectional online survey of adult MSM in Lima, Perú to evaluate patterns of SOPV attendance, associated sexual risk behaviors, and attitudes toward SOPV-based interventions. Participants were recruited through outreach to social media networks affiliated with local LGBTQ-aligned community groups. Our primary analytic objective was to estimate the association of HIV-related sexual risk behaviors and SOPV attendance. Additionally, we performed exploratory analyses to describe risk behavior stratified by SOPV category and to examine the relationship between SOPV attendance and the use of online platforms to meet sex partners.

**Results:**

Overall, 389 MSM completed the survey from November 2018 through May 2019, of whom 68% reported attending an SOPV in the last 3 months. SOPV attendance was associated with multiple sexual risk behaviors, including transactional sex, group sex, substance use around the time of sex, and higher number of partners. Over two thirds of SOPV attendees indicated they would accept HIV testing if offered at SOPVs.

**Conclusions:**

SOPV attendance was common among MSM in Lima who participated in our survey, and SOPV attendees reported significantly greater engagement in sexual risk behaviors related to HIV transmission. Attitudes toward hypothetical SOPV-based interventions were generally favorable. These findings suggest that outreach at SOPVs may be an effective mechanism for reaching a particularly high-risk sub-population of MSM in Perú to deliver targeted HIV testing and prevention interventions.

## Background

As in most of the Americas, the HIV epidemic in Perú is concentrated among men who have sex with men (MSM) and transgender women (TW). Prevalence among these groups countrywide in 2018 was reported to be 12 and 16%, respectively [1], although in the capital city of Lima it may be substantially higher, with estimates ranging from 18-20% among MSM and 17-30% among TW [2–6]. In addition, high HIV incidence persists, with some studies reporting rates of >10 per 100 person-years [3, 4, 7]. Most notable, however, is the fact that HIV incidence in Perú has not significantly changed over the last decade – despite the broad scale-up of antiretroviral treatment (ART) access during this time. While UNAIDS estimates suggest a stable overall incidence rate of 1.1 (confidence interval [CI] 0.7 – 1.8) per 100 person-years in 2010 compared to 0.9 (CI 0.5 – 1.6) in 2017 [1], data from the Peruvian Ministry of Health show a 27% increase (from 4677 to 5926 cases) in new HIV diagnoses over this same time period [8]. Since 2004 the Ministry of Health has provided antiretroviral treatment (ART) at no cost to individuals with HIV infection who meet clinical and immunologic criteria [9], and has moved to extend ART coverage to all Peruvians living with HIV following the release of the World Health Organization (WHO) “treat all” recommendations in 2015 [10–12]. In contrast to the expansion in access to ART for HIV treatment, the use of antiretroviral drugs for pre-exposure prophylaxis (PrEP) is not yet subsidized in Perú and access remains quite limited, with fewer than 1500 active PrEP users estimated in the country currently [13].

Delayed diagnosis of HIV infection is an important factor fueling onward sexual transmission in Perú [14–17]. One recent study estimated that just 24% of MSM and TW living with HIV in Perú have been diagnosed, and among these only half are linked to care and on ART [18] – falling far short of the UNAIDS “90-90-90” targets [19]. To close these gaps in Perú’s HIV services continuum, new approaches are needed that more effectively engage vulnerable MSM and TW. Strategies that aim to address the underlying psychological and structural barriers experienced by members of these communities – including stigma, fear, low risk perception, and geographic factors [20–23] – are most likely to have success.

Previous research suggests that offering HIV testing at community-based venues can reach individuals who may not otherwise access traditional clinic-based services [24–28], including in Perú [5, 7, 29]. Sex-on-premise venues (SOPVs), which are social venues that provide a space to meet and have sex with other patrons, may be fitting sites for such outreach. Although sex is the primary, and often overtly stated, purpose of many SOPVs (e.g. sex clubs, pornographic movie theaters, and most saunas/bathhouses), the term also encompasses commercial establishments where sex is permitted or encouraged, even if not explicitly endorsed as its reason for being (e.g. hourly hotels and bars/discos with dark rooms) [30, 31]. Studies from high-income countries (HIC) with concentrated HIV epidemics affecting MSM, including Australia and the United States, have found an association between SOPV attendance and high-risk sexual behavior [32–36]. In addition, the feasibility of delivering sexual health interventions at SOPVs has been well documented in HIC settings, including programs offering safer sex counseling, free condom distribution, on-site testing for HIV and other sexually transmitted infections (STI), and distribution of HIV self-testing kits [37–47]. While intervention uptake among SOPV patrons was relatively modest in two studies that assessed this outcome (uptake was 24% [37] and 51% [39], respectively), several studies demonstrated SOPV-based HIV/STI testing to be effective at reaching individuals at high risk, including those who may not otherwise come to be tested in healthcare facilities. In contrast to the evidence base from HICs, research related to SOPVs and the populations who attend them in low- and middle-income countries (LMIC) such as Perú remains extremely limited.

Despite the paucity of evidence on the role of SOPVs in Perú’s HIV epidemic – and in the Latin American context more broadly – several factors suggest that SOPVs may be ideal sites at which to conduct outreach to Peruvian MSM and TW at highest risk for HIV infection. MSM and TW in Perú commonly live at home with their family of origin [48, 49], where conservative social norms [50] and stigma related to gender and sexuality [51–53] can create powerful disincentives to bringing sex partners home. By functioning as alternative locations where MSM and TW can go to meet sex partners, SOPVs may play a fundamentally different – and relatively more important – role in Perú as compared to regions with more progressive social norms, including the HIC settings where much of the existing literature on SOPVs is based. Additionally, the use of online platforms to facilitate efficient identification of sex partners – including geosocial networking apps – is increasing in Perú [49]. SOPVs may be important meeting places for sex after meeting a prospective partner online. Importantly, SOPVs are by definition physical locations where individuals gather. Thus, in contrast to internet-based outreach, SOPVs are sites where HIV testing and biomedical prevention interventions could be delivered directly. Several recent studies among MSM and TW in Lima demonstrated high uptake of HIV testing, as well as high rates of new HIV diagnoses, at bars and other public social venues – including some SOPVs [5, 7, 29]. However, we were unable to identify any dedicated research from Perú – or any other Latin American country – focusing specifically on SOPVs or their clientele. One possible explanation is that SOPVs are only now being appreciated, and their importance emphasized, in the context of the surging popularity of online platforms to meet sex partners. In order to better understand the role of SOPVs within high-risk sexual networks in Perú, and to inform future SOPV-based outreach strategies to deliver HIV testing and prevention interventions, we conducted an online survey of MSM and TW in Lima. The goals of this study were to evaluate the prevalence and patterns of SOPV attendance, the association of SOPV attendance with sexual risk behaviors, and the attitudes toward potential SOPV-based interventions in these communities.

## Methods

### Study population and design

We conducted a cross-sectional, internet-based survey among MSM and TW in Lima, Perú. Adults 18 years of age or greater who identified as either MSM or TW were eligible. We recruited participants by disseminating a link to the survey via social media platforms affiliated with a local LGBTQ-aligned community-based organization in Lima. An initial landing page included the consent form, basic instructions, and eligibility criteria. This was formatted for both desktop and mobile access, enabling survey participation from any computer or mobile device with an internet connection. Prior to accessing the survey, individuals were required to provide an electronic signature as an attestation of their eligibility and informed consent. Participation was completely anonymous and no incentive was offered. The study underwent bioethics review and received approvals from both the Vía Libre Comité Institucional de Bioética (Lima, Perú) and the University of Washington Institutional Review Board (Seattle, USA).

### Survey instrument

REDCap [54] was used for survey instrument development and administration, as well as data collection and storage. The survey included a total of 19 main questions, some of which branched to sub-questions based on the response (Additional file 1). Depending on the number of sub-questions prompted (based on answers to the main questions), survey completion took approximately 10-15 min. We collected basic demographic data and asked participants about venue attendance and sexual behaviors in the past 3 months, including how/where they met their recent partners and where they went to have sex, as well as online platforms used to meet partners. In addition to questions about SOPV attendance and sexual behaviors in general, we collected more detailed venue-specific data for participants who reported sex at an SOPV with either their last or penultimate partner (in the last 3 months). This included information about the physical and environmental features of the venue(s) they attended, sexual behaviors there (both observed and participated, including condom use), and attitudes toward hypothetical venue-based sexual health interventions such as condom/lubricant distribution and point-of-care testing for HIV and other sexually transmitted infections (STIs).

### Statistical analysis

After reaching our target of 400 completed surveys, data were exported from REDCap into Stata version 15 (StataCorp. 2017. *Stata Statistical Software: Release 15*. College Station, TX: StataCorp LLC) for analysis. We used descriptive statistics to summarize the proportion of participants who reported attending an SOPV in the last 3 months, as well as participant characteristics, including demographics, sexual behaviors (including the overall proportion who used an online platform to meet a sex partner in the last 3 months), knowledge of biomedical HIV prevention (e.g. PrEP, “U=U”), attitudes toward venue-based HIV testing and prevention interventions, and other venue-specific factors. The objective of our primary analysis was to evaluate the relationship between sexual risk behaviors and SOPV attendance in the previous 3 months. We defined an SOPV as any of the following 5 venue categories: sauna, hotel, sex club, pornographic movie theater, or bar/disco known to permit sex. Additionally, we defined two categories of SOPV attendance: *meeting* a partner at, and *having sex* at, an SOPV. We estimated the crude prevalence ratio (PR) and 95% confidence interval (CI) for each factor of interest (all of which were coded as dichotomous variables), comparing the proportion with a given factor among participants who reported meeting a partner at (or having sex at) an SOPV in the previous 3 months to the proportion among those who did not. To further examine patterns of SOPV attendance and use of online platforms to identify sex partners, we conducted several exploratory sub-analyses, stratifying by SOPV category, using the detailed venue-level data collected on the last two partners. All statistical testing to estimate confidence intervals and prevalence ratios used the Chi-squared distribution. Statistical testing to assess for differences in proportions used either the Chi-squared or Fisher’s exact test.

## Results

From November 26, 2018 through May 16, 2019 a total of 389 MSM and 8 TW completed the online survey. Two transgender male and two cisgender female identifying individuals also completed a survey but were excluded from the analysis as they did not meet predetermined inclusion criteria. Additionally, because the low number of TW participants was insufficient to draw meaningful inferences for this important but distinct sub-population [55], we restricted our analysis to cisgender MSM only.

### Demographic and behavioral characteristics

Among 389 MSM, 77% identified as homosexual, 19% as bisexual, and 3% as heterosexual (Table 1). Median age was 30 years (interquartile range [IQR] 25 – 37), and just under half (47%) had a university degree or higher (likely a reflection of the social media sites used for recruitment). Over three quarters lived with at least one family member, and more than half lived with one or both parents. Participants reported a median of 3 total sex partners (IQR 2 – 5) in the last 3 months. Nearly half reported sex under the influence of either drugs or alcohol, about one third participated in group sex, and about a quarter reported transactional sex (either received payment or paid for sex); however, only 2% identified as a sex worker. Overall, 85% reported ever being tested for HIV and 26% reported being HIV positive, the vast majority (94%) of whom indicated they were taking ART. When given a statement illustrating the concept of “U=U” (i.e. that sexual transmission of HIV is virtually impossible from an HIV-infected person with an undetectable viral load on ART), roughly half (53%) indicated this was true. A somewhat larger proportion (73%) reported that they had ever heard of PrEP.

**Table 1:** Demographic Characteristics, Sexual Behavior, and HIV Testing History (*N* = 389)

**Panel A. Demographic Characteristics**		**Panel B. Sexual Behaviors**	
	Median (IQR)		n (% of N)
Age (years)	30 (25 – 37)	≥1 sex partner	365 (94%)
Monthly income (Soles)^a^	1500 (700 – 3000)	Had “casual” partner^d^	215 (55%)
		Group sex	141 (36%)
	n (% of N)	Transactional sex (any)	110 (28%)
Highest Education		Paid for sex	67 (17%)
Secondary school or less	152 (39%)	Was paid for sex	57 (15%)
Technical/Vocational	56 (14%)	Identifies as sex worker	8 (2%)
University degree or more	181 (47%)	Substance use associated w/sex (any)	172 (44%)
Living Situation^b^		Alcohol	144 (37%)
Lives alone	62 (16%)	Marijuana	66 (17%)
With a friend / roommate	34 (9%)	Poppers	47 (12%)
With spouse / stable partner	56 (14%)	Other^e^	10 (3%)
With any family member^c^	301 (77%)	Condomless anal sex (receptive OR insertive)^d^	176 (45%)
Sexual Identity/Orientation		Receptive	121 (31%)
Homosexual	299 (77%)	Insertive	118 (30%)
Bisexual	74 (19%)	HIV Prevention Knowledge	
Heterosexual	13 (3%)	Ever heard of PrEP	285 (73%)
Pansexual	3 (< 1%)	Believes that “U=U” is true^f^	208 (53%)
**Panel C. HIV Testing History**			
	n (% of N)		n (% of *N* = 227 HIV-)
HIV-positive	102 (26%)	Last negative HIV test	
On ART, n (% of *N* = 102 HIV+)	96 (94%)	< 3 months ago	89 (39%)
Unknown HIV status / Never tested	49 (13%)	3-6 months ago	54 (24%)
Declined to disclose HIV status	11 (3%)	6-12 months ago	42 (19%)
HIV-negative when last tested	227 (58%)	≥12 months ago	41 (18%)

*MSM* men who have sex with men, *IQR* interquartile range, *ART* antiretroviral treatment, *PrEP* pre-exposure prophylaxis; ^a^Exchange rate on 26 Nov 2018: 1 USD = 3.37 Peruvian Nuevo Soles [56]; ^b^Categories not mutually exclusive; ^c^Specific family members: Mother 190 (49%); Father 129 (33%); Sibling 166 (43%); Aunt/Uncle 50 (13%); Cousin 34 (9%); Grandparent 31 (8%); Niece/Nephew 5 (1%); Son/Daughter 3 (< 1%); Brother-in-Law/Sister-in-Law 3 (< 1%); ^d^Refers to last or penultimate partner; ^e^Other drugs: Cocaine 5 (1%); Ecstasy 4 (1%); Amphetamine 2 (< 1%); Heroin 1 (< 1%); “Eme” (< 1%); ^f^Responded “true” to the following statement: “*It is very unlikely that a person with HIV will transmit the virus to their sexual partner if the person with HIV is taking antiretroviral therapy and the virus is undetectable in their blood*”

### Prevalence of SOPV attendance and online platform use to meet sex partners

Recent SOPV attendance was common, as was the use of online platforms to meet a sex partner (Table 2). Overall, 68% reported either meeting a partner or having sex at an SOPV at least once in the last 3 months (42% *met* a partner at an SOPV and 61% *had sex* at an SOPV). The most common SOPV category for *meeting* a new partner was bars/discos, while the most common SOPV category for *having sex* was hotels. The use of online platforms to meet sex partners was also common: overall, 78% reported meeting a partner online in the last 3 months (median 3 online partners, IQR 1 – 5). Grindr was by far the most frequently reported online platform, followed by Facebook and WhatsApp.

**Table 2:** SOPV Attendance and Online Platform Use (*N* = 389)

**Panel A. SOPVs Attended to Meet a Partner or Have Sex in Last 3 Months**			
	n (% of N)		
	MET Partner ***or*** Had SEX at SOPV	MET Partner at SOPV	Had SEX at SOPV
Any SOPV	264 (68%)	165 (42%)	236 (61%)
Hotel	193 (50%)	3 (< 1%)	193 (50%)
Bar/Disco	109 (28%)	95 (24%)	34 (9%)
Sauna	98 (25%)	90 (23%)	84 (22%)
Sex Club	40 (10%)	36 (9%)	29 (7%)
Porno Theater	18 (5%)	15 (4%)	15 (4%)
**Panel B. Online Platforms Used to Meet a Sex Partner in Last 3 Months**			
	n (% of N)		
Met ≥1 partner online (any platform)	304 (78%)		
Grindr	217 (56%)		
Facebook	84 (22%)		
WhatsApp group	42 (11%)		
Manhunt	28 (7%)		
Other^a^	72 (19%)		

*SOPV* sex-on-premise venue; ^a^Other online platforms, n (%): Tinder 20 (5%); Scruff 18 (5%); “Chat” (e.g. PeruGayChat, GayChat, ChatGay, ChatPeruGay, ElChat) 10 (3%); Instagram 8 (2%); GayRomeo 1 (< 1%); Surge 1 (< 1%)

### Association of SOPV attendance and sexual risk characteristics

SOPV attendees, as compared to MSM who did not attend an SOPV in the last 3 months, were more likely to report several sexual risk behaviors, including group sex, transactional sex, sex under the influence of alcohol, sex with a “casual” partner, and ≥ 3 sex partners in the last 3 months; these associations were statistically significant in all cases regardless of whether evaluated with respect to *meeting* a partner or to *having sex* at an SOPV (Table 3). SOPV attendees were somewhat more likely to report having ever been tested for HIV. However, self-reported HIV seropositivity was not associated with SOPV attendance. *Meeting* a partner at an SOPV, but not *having sex* at an SOPV, was associated with older age and higher monthly income. *Having sex* at an SOPV, but not *meeting* a partner at an SOPV, was associated with living with family and with having a recent online partner. We performed sensitivity analyses excluding self-reported HIV-positive individuals, which did not substantively alter our findings regarding the relationship between SOPV attendance and sexual risk behaviors.

**Table 3:** Factors Associated with SOPV Attendance in the Last 3 Months

	% of N	Prevalence Ratio (95% CI)	
	Overall Prevalence (N = 389)	MET a partner ≥1X at SOPV (*n* = 165)	Had SEX ≥1X at SOPV (*n* = 236)
**Demographic Characteristics**			
Age ≥ 30 years	51%	**1.28 (1.05 – 1.55)**	1.11 (0.90 – 1.36)
Bisexual identifying	19%	0.93 (0.61 – 1.41)	0.90 (0.59 – 1.36)
Monthly income ≥1500 Soles^a^	52%	**1.21 (1.00 – 1.46)**	1.12 (0.91 – 1.36)
Lives with any family member	77%	1.03 (0.93 – 1.15)	**1.16 (1.03 – 1.30)**
University education	47%	1.08 (0.87 – 1.33)	1.05 (0.84 – 1.31)
**Sexual Behaviors in Last 3 Months**			
≥ 3 sex partners	54%	**2.00 (1.65 – 2.41)**	**1.92 (1.52 – 2.43)**
Met ≥1 partner online	78%	0.96 (0.86 – 1.07)	**1.13 (1.01 – 1.26)**
Group sex	36%	**2.12 (1.62 – 2.79)**	**1.70 (1.25 – 2.31)**
Transactional sex	28%	**1.63 (1.19 – 2.24)**	**2.45 (1.62 – 3.70)**
Had “casual” partner^b^	55%	**1.42 (1.19 – 1.69)**	**1.50 (1.21 – 1.84)**
Condomless anal sex^b^	45%	1.18 (0.95 – 1.47)	1.19 (0.94 – 1.50)
Substance use associated w/sex (any)	44%	**1.64 (1.31 – 2.04)**	**1.67 (1.29 – 2.18)**
Alcohol	37%	**1.65 (1.27 – 2.14)**	**1.69 (1.25 – 2.28)**
Marijuana	17%	**1.73 (1.11 – 2.70)**	1.21 (0.76 – 1.93)
Poppers	12%	**2.90 (1.62 – 5.17)**	**2.74 (1.36 – 5.50)**
**HIV Testing History**			
Ever had an HIV test	85%	**1.09 (1.01 – 1.18)**	**1.11 (1.02 – 1.22)**
HIV positive	26%	1.36 (0.97 – 1.89)	0.96 (0.69 – 1.35)

Statistically significant associations (*p* < 0.05) denoted in bold; *SOPV* sex-on-premise venue; ^a^Exchange rate on 26 Nov 2018: 1 USD = 3.37 Peruvian Nuevo Soles [56]; ^b^Refers to last or penultimate partner.

### Sexual Risk Behaviors and Online Platform Use Stratified by SOPV Category

Stratification by SOPV category revealed heterogeneity in the sexual risk behaviors reported by populations of MSM as defined by attendance of different categories of venue to either *meet* a partner or *have sex* (Table 4). For this exploratory sub-analysis, we define SOPV attendance as either *meeting* a partner or *having sex* at a given type of SOPV and we report only descriptive statistics, as participants could indicate they attended multiple venues, precluding the use of inferential statistical tests.

**Table 4:** Sexual Risk Behaviors and HIV Status Stratified by SOPV Category Attended in the Last 3 Months

Risk Characteristic^**a**^	Category of SOPV Attended in Last 3 Months, n (% of N)					
	Any SOPV (*N* = 264)	Hotel (*N* = 193)	Sauna (*N* = 98)	Sex Club (*N* = 40)	Porno Theater (*N* = 18)	Bar/Disco (*N* = 109)
≥3 sex partners	172 (65%)	124 (65%)	83 (85%)	37 (93%)	18 (100%)	75 (69%)
Group sex	111 (42%)	74 (38%)	59 (60%)	28 (70%)	13 (72%)	54 (50%)
Transactional sex	91 (34%)	70 (36%)	34 (35%)	21 (53%)	12 (67%)	38 (35%)
“Casual” partner^b^	165 (63%)	119 (62%)	69 (70%)	30 (75%)	13 (72%)	70 (64%)
Condomless anal sex^b^	126 (48%)	93 (48%)	52 (53%)	15 (38%)	10 (56%)	54 (50%)
Substance use w/sex	142 (54%)	102 (53%)	52 (53%)	24 (60%)	8 (44%)	68 (62%)
HIV positive	69 (26%)	42 (22%)	33 (34%)	16 (40%)	7 (39%)	33 (30%)

SOPV attendance is defined as either *meeting* a partner or *having sex* at a given SOPV type in the last 3 months; categories (columns) are not mutually exclusive. *SOPV* sex-on-premise venue; ^a^Refers to within the last 3 months (where applicable); ^b^Refers to last or penultimate partner

Among participants who *met* their last or penultimate partner online, we tabulated the SOPV categories they attended to *have sex* with that same partner. Of 241 participants who met their *last* partner online, 66 (27%) had sex at a hotel, 4 (2%) at a sauna, and 2 (< 1%) at a bar or disco; none had sex at a porno theater or sex club. Of 173 participants who met their *penultimate* partner online, 54 (31%) had sex at a hotel, 2 (1%) at a sauna, 1 (< 1%) at a sex club, and 1 (< 1%) at a bar/disco; none had sex at a porno theater. Overall, compared to those who did *not* meet a partner online, participants who met *any* recent partner online were more likely to report sex at a hotel (54% among those with a recent online partner vs 34% among those with no recent online partner, *p* = 0.001) or at a bar/disco (11% vs 2%, *p* = 0.016) in the last 3 months, but not at a sauna (22% vs 21%, *p* = 0.916), sex club (8% vs 6%, *p* = 0.532), or porno theater (4% vs 5%, *p* = 0.749).

### Sexual risk at SOPVs and attitudes toward SOPV-based interventions

**Fig. 1:**
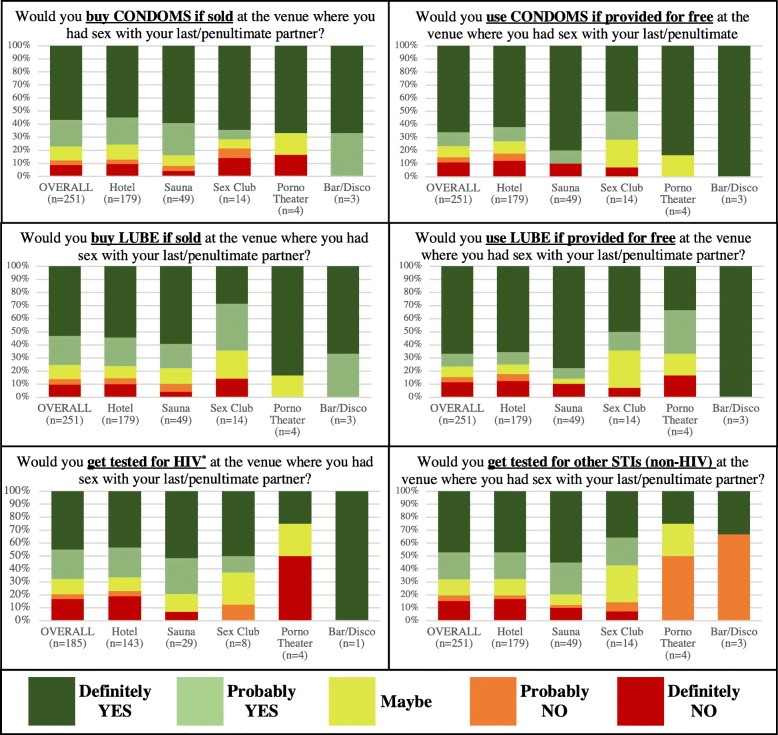
Attitudes Toward Hypothetical SOPV-Based HIV/STI Testing and Sexual Health Interventions. Venue-level data are based on an aggregate total of *n* = 251 responses corresponding to the SOPVs where a total of *N* = 177 MSM reported having a sexual encounter with their last (*n* = 138) and/or penultimate (*n* = 113) partner in the last 3 months. *SOPV* sex-on-premise venue; *STI* sexually transmitted infection. ^*^Excludes participants who self-report being HIV positive

For MSM who had sex at an SOPV with at least one of their last two partners (N = 177), we tabulated venue-specific data on sexual encounters with their last (n = 138) and/or penultimate (n = 113) partners. Participants indicated that condoms were available when they had a sexual encounter at an SOPV 49% of the time overall, including 48% of the time at hotels, 57% at saunas, 76% at sex clubs, 25% at porno theaters, and 0% at bars/discos. Lube was available at the SOPV for 27% of the encounters (29% at hotels, 29% at saunas, 13% at sex clubs, 0% at porno theaters, and 50% at bars/discos). Alcohol was present at the SOPV 29% of the time for SOPVs overall (24% at hotels, 43% at saunas, 75% at sex clubs, 0% at porno theaters, and 50% at bars/discos) and group sex occurred at 20% of encounters (12% at hotels, 33% at saunas, 75% at sex clubs, 50% at porno theaters, and 50% at bars/discos). Attitudes toward hypothetical SOPV-based sexual health interventions, such as HIV/STI testing and condom/lube distribution, varied based on the category of SOPV in question, but generally indicated a high level of acceptability (Fig. 1).

## Discussion

More than two thirds of our study population attended an SOPV either to meet a sex partner or to have sex in the last 3 months, suggesting that SOPV attendance may be quite common among MSM in Lima. In addition, those who attended an SOPV were significantly more likely to report behaviors associated with elevated risk of HIV and STI transmission, including group sex, transactional sex, sex under the influence of alcohol, sex with a casual partner, and more overall partners. Notably, the majority of SOPV attendees indicated favorable attitudes toward theoretical SOPV-based sexual health interventions such as condom/lubricant distribution and HIV/STI testing.

Although SOPV attendance was associated with several sexual risk behaviors, one notable exception was condomless anal sex. One possible explanation is that we asked questions about sexual positioning and condom use with reference to only the last two partners, rather than asking about *all* recent partners (as was asked for other behaviors). However, similar results to ours were observed in a large online survey conducted in the mid-2000s of MSM in the US, which found that meeting a partner at a physical venue (e.g. bars/clubs, bathhouses, and public outdoor spaces) was associated with risk behaviors such as alcohol use – but not with condomless anal sex [33]. In our exploratory analyses, sexual risk behavior also appeared to vary by SOPV category. For example, compared with other categories, the proportion of MSM reporting nearly every risk characteristic (except condomless anal sex) was higher for sex clubs (Table 4). In contrast, hourly hotels, which were by far the most common SOPV attended, appeared to be frequented by lower risk clientele. Taken together, these results suggest that more nuanced data collection may be necessary to characterize sexual risk behaviors associated with SOPV attendance in this setting. Such information, including the identification of specific SOPVs attended by the highest risk clientele, would be particularly germane to the development of SOPV-based outreach strategies to deliver HIV testing and other sexual health interventions.

The vast majority (78%) of our sample population reported meeting a sex partner online in the last 3 months. This is consistent with observations from a large contemporaneous online survey of MSM in Brazil, Mexico, and Perú conducted in 2018 by Torres et al., which found that 81% of Peruvian MSM reported using apps for sexual encounters [57]. Notably, these estimates are roughly double what was found in two studies of MSM in Lima from just a few years ago, including one conducted in 2013-2014 (in person) and another in 2012-2013 (online), which reported 37 and 44% of participants had a recent online partner, respectively [3, 49]. Together these findings likely reflect the rapid expansion in recent years of access to low-cost internet services and mobile devices in Perú; however, the use of an online survey, which selects for individuals with internet access, may have also played a small role in the higher estimates observed in both our study and by Torres et al. [57]. In addition, geosocial networking applications (e.g. Grindr), which have been associated with increased sexual risk behavior and STI incidence in some settings [58–60], have had substantial growth in popularity since the two earlier studies.

Our results also shed light on the relationship between online platform use and SOPV attendance. MSM who *had sex* at an SOPV, but not those who *met* a partner at an SOPV, were significantly more likely to report meeting a recent partner online. This mirrored what we observed for one’s living situation: living with family was associated *having sex* at an SOPV but not *meeting* a partner at one. One potential explanation for this pattern is that, although online platforms can facilitate the *identification* of sex partners, stigma precludes those who live with family from having sex with these partners at home. SOPVs might fill this void by offering places outside of the home where MSM can have sex with online partners. We also found that, when stratified by SOPV category, the association between SOPV attendance and online platform use was significant only for hotels and bars/discos, suggesting that these venues may be preferred rendezvous sites for meeting online partners for sex, perhaps because identifying new sex partners in person may be easier at saunas, sex clubs, and porno theaters.

The primary limitation of this study relates to its generalizability. Our sampling method, which relied on recruitment through local social media networks, selected a population that is somewhat more educated and affluent than the general population of MSM in Lima. Therefore, our estimates of SOPV attendance and sexual behavior may not reflect the experiences of MSM in lower socioeconomic strata. Self-reported HIV prevalence was 26%, which is within the range reported by other studies of MSM in Lima [5, 6]. However, over 80% of MSM in this study reported having ever received an HIV test and over 90% of those who self-reported being HIV positive indicated they were taking ART. In both cases, these rates are somewhat higher than past population estimates of HIV testing and ART coverage, respectively, in the region [1, 18], suggesting above average access to health services. If so, the estimates derived from our sample population may in fact under-represent the risk profile of MSM in Lima on average. Future studies of SOPV attendance and associated sexual risk behaviors in Lima should consider utilizing alternative sampling strategies to include MSM in less affluent communities, as well as TW – an extremely vulnerable population that our online recruitment strategy did not adequately reach.

Although, overall, participants reported favorable attitudes toward SOPV-based HIV/STI testing interventions (Fig. 1), interpretation of these data is limited by the small sample size for sex clubs, porno theaters, and bars/discos. The use of a survey to evaluate acceptability of hypothetical interventions may also overestimate uptake in real-world settings. Nevertheless, our results, which indicate that over two thirds of MSM would either “definitely” (45%) or “probably” (23%) accept SOPV-based HIV testing, are consistent with direct observations from the field, including a recent study that demonstrated 52% uptake of venue-based HIV testing when it was offered to MSM and TW at bars, clubs, and public parks in Lima [5].

Despite the limitations to its generalizability, the online administration of our survey allowed it to be conducted in a completely anonymous fashion. This significantly reduced the likelihood of social desirability bias and is an important strength of the study. While anonymous participation can also, in theory, enable a single person to complete more than one survey, there was no incentive to do so and completion of the survey was relatively time-intensive (~ 10-15 min on average). Therefore, duplicate survey responses or other spurious results related to external incentives are unlikely.

In summary, our results support the suggestion that SOPVs play an important role in MSM sexual networks in Perú. This may be particularly pronounced compared to settings where young adults tend to leave the family home at a younger age, or where stigma is a less prominent driver of sexual decision-making. Both SOPV attendance and the use of online platforms to meet sex partners appear to be highly prevalent behaviors among MSM in Lima. Furthermore, our findings indicate that MSM who attend SOPVs may be at particularly high risk for HIV/STI transmission, underscoring an opportunity to work with SOPVs to develop interventions that will expand access to testing, treatment, and prevention services. Although access to PrEP is relatively limited at present time in Perú, as it becomes more available partnerships with SOPVs could be leveraged to promote linkage to PrEP providers or even facilitate medication distribution. Importantly, our results suggest SOPV-based interventions would be accepted by most MSM in Lima who frequent these venues. Overall, these findings have important public health implications and suggest that SOPVs in Lima are suitable sites for targeted HIV testing and prevention interventions.

## Conclusions

Among MSM in Lima, SOPV attendance appears to be common and SOPV attendees may be at higher risk for HIV transmission compared to MSM in general. SOPV-based outreach may be an acceptable strategy to deliver sexual health interventions in this community, and SOPVs may be ideal points of contact where MSM in high-risk sexual networks – including those who meet partners online – could be reached for HIV testing. Our findings support further development of targeted SOPV-based interventions as a way to increase access to HIV testing, treatment, and prevention services for MSM in Perú.

## Supplementary information

**Additional file 1.** (AdditionalFile1.pdf): Annotated Survey Instrument. Full-length Spanish-language text of survey instrument used in the study, including transitional text and instructional notes used to guide participants, as well as annotations indicating branching logic; all items are in the same order as they appeared to participants taking the actual online survey in REDCap.

## Data Availability

The datasets used and analyzed during the current study are available from the corresponding author on reasonable request.

## Abbreviations

MSM: Men who have sex with men
SOPV: Sex-on-premise venue
TW: Transgender women
WHO: World Health Organization
ART: Antiretroviral treatment
PrEP: Pre-exposure prophylaxis
STI: Sexually transmitted infection
HIC: High-income country
LMIC: Low/middle-income country
PR: Prevalence ratio
CI: Confidence interval
IQR: Interquartile range
U=U: Undetectable equals untransmissible
