# Supplementary material for: Sex-on-premise venues, associated risk behaviors, and attitudes toward venue-based HIV testing among men who have sex with men in Lima, Perú
Source: BMC Public Health. 2020 Apr 19;20:521. doi: 10.1186/s12889-020-08604-w (PMC7168867; doi:10.1186/s12889-020-08604-w)
Supplement: Supplementary file 1 — Additional file 1. (AdditionalFile1.pdf): Annotated Survey Instrument. Full-length Spanish-language text of survey instrument used in the study, including transitional text and instructional notes used to guide participants, as well as annotations indicating branching logic; all items are in the same order as they appeared to participants taking the actual online survey in REDCap. [file 12889_2020_8604_MOESM1_ESM.pdf]

SURVEY INSTRUMENT

1. ¿Cuantos años tiene usted?
   Marque su edad
   - ______

2. ¿Cómo se considera respecto a su género?
   Marque sólo una opción
   a. Hombre
   b. Mujer transgénero, travesti, o transexual
   c. Hombre transgénero
   d. Otro ________ (*indique cómo se considera*)

3. ¿Cómo se considera respecto a su sexualidad?
   Marque sólo una opción
   a. Homosexual
   b. Bisexual
   c. Heterosexual
   d. Otro ________ (*indique cómo se considera*)

4. ¿Cuál es el nivel más alto de educación que ha logrado?
   Marque sólo una opción
   a. Ninguno
   b. Escuela primaria incompleta
   c. Escuela primaria completa
   d. Escuela secundaria incompleta
   e. Escuela secundaria completa
   f. Educación superior técnica incompleta
   g. Educación superior técnica completa
   h. Educación universitaria incompleta
   i. Educación universitaria completa
   j. Educación post-grado (p.ej. maestría o doctoral)
   k. Otro ________ (*indique cual*)

5. Aproximadamente, ¿Cuánto fue su ingreso económico total en el último mes?
   a. No tuve ningún ingreso
   b. Tuve ingreso de ________ Nuevos Soles (*escriba su mejor estimado*)

6. ¿Con quién vive?
   Marque todas las opciones que apliquen
   a. Padre
   b. Madre
   c. Hermano(s)
   d. Hermana(s)
   e. Abuelo/a(s)

f. Tío/a(s)
g. Primo/a(s)
h. Amigo/a(s) y/o otros compañero/a(s) de cuarto
i. Pareja estable
j. Vivo solo
k. Otra(s) persona(s) ________ (*indícala(s)*)

LAS SIGUIENTES PREGUNTAS SON ACERCA DE LOS TIPOS DE PAREJAS SEXUALES QUE HA CONOCIDO EN LOS ÙLTIMOS 3 MESES, EN QUE LUGARES LOS HA CONOCIDO Y A QUE LUGARES SE HAN IDO A TENER RELACIONES SEXUALES.

AHORA, PIENSE EN <u>COMO CONOCIÓ</u> A SUS ULTIMAS PAREJAS SEXUALES.

7. En los últimos **<u>3 MESES</u>**, ¿alguna vez conoció a una pareja sexual en línea (por ejemplo, páginas del Internet o aplicaciones de citas)? De responder sí, ¿cuántas parejas sexuales conoció en línea (en los últimos 3 meses)?
    a. No
    b. Sí ____________ *(escriba cuántas)*

    De ser sí, ¿En cuales páginas del Internet o apps conoció a una nueva pareja sexual (en los últimos 3 meses)?
    Marque todas las opciones que apliquen
    - Facebook
    - Grindr
    - Manhunt
    - Gayromeo
    - Hornet
    - PlanetRomeo
    - Surge
    - Scruff
    - Grupos de WhatsApp
    - Otras páginas o apps: _________ *(escriba los nombres)*

8. En los últimos **<u>3 MESES</u>**, ¿alguna vez conoció a una pareja sexual en un espacio público o lugar social? *Puede ser cualquier de los siguientes tipos de lugares: disco, bar, plaza, calle, parque, playa, centro comercial, sauna, sala de cine, club sexual o sala de videos, cabinas de internet, peluquería, gimnasio / campo atlético, baño público, o fiesta privada en la casa de alguien. Recuerde que esta pregunta trata del lugar donde <u>conoció</u> a una pareja sexual, pudiendo ser también el mismo lugar en donde tuvieron sexo o no.*
    a. No, no conocí a ninguna pareja sexual en estos tipos de lugares
    b. Sí, conocí a una pareja sexual (o más) en uno de estos tipos de lugares

    De ser sí, ¿en cuáles de los siguientes lugares conoció a una pareja con quien tuvo sexo después? *Para cada tipo de lugar que indica, también indique el número de parejas que conoció allí en los últimos <u>3 meses</u>.*
    Marque todas las opciones que apliquen
    - Disco ___ *(¿cuantas en los últimos 3 meses?)*
    - Bar ___ *(¿cuantas en los últimos 3 meses?)*
    - Plaza o Calle___ *(¿cuantas en los últimos 3 meses?)*

- Parque ___ *(¿cuantas en los últimos 3 meses?)*
- Playa ___ *(¿cuantas en los últimos 3 meses?)*
- Centro comercial ___ *(¿cuantas en los últimos 3 meses?)*
- Sauna ___ *(¿cuantas en los últimos 3 meses?)*
- Sala de cine para adultos ___ *(¿cuantas en los últimos 3 meses?)*
- Club sexual o Sala de videos ___ *(¿cuantas en los últimos 3 meses?)*
- Cabinas de Internet ___ *(¿cuantas en los últimos 3 meses?)*
- Peluquería ___ *(¿cuantas en los últimos 3 meses?)*
- Gimnasio o Campo atlético ___ *(¿cuantas en los últimos 3 meses?)*
- Baño publico ___ *(¿cuantas en los últimos 3 meses?)*
- Fiesta privada (en una casa/apartamento) ___ *(¿cuantas en los últimos 3 meses?)*
- Otro tipo de espacio público o lugar social ___ *(¿cuantas en los últimos 3 meses)*

AHORA, PIENSE EN LOS LUGARES ESPECIFICOS DONDE <u>TUVO SEXO</u>, PUDIENDO SER EL MISMO LUGAR DONDE CONOCIÓ A UNA PAREJA O NO.

9. En los últimos **<u>3 MESES</u>**, ¿alguna vez tuvo sexo en uno de los siguientes tipos de lugares? *Puede ser cualquier de los siguientes tipos de lugares: disco, bar, plaza, calle, parque, playa, centro comercial, hotel, sauna, sala de cine, club sexual o sala de videos, cabinas de internet, peluquería, gimnasio / campo atlético, baño público, o fiesta privada en la casa de alguien, casa de travestis, su casa, la casa de una pareja sexual.*
    a. No, no tuve sexo en los últimos 3 meses
    b. Sí, tuve sexo en uno (o más) de los siguientes lugares

De ser sí, indique cuántas veces tuvo sexo en cada uno de los siguientes lugares.
Marque todas las opciones que apliquen

- Cuarto oscuro/privado de un bar o disco ___ *(¿cuantas veces en los últimos 3 meses?)*
- Espacio afuera (p.ej. plaza, calle, parque, playa) ___ *(¿cuantas veces en los últimos 3 meses?)*
- Centro comercial ___ *(¿cuantas veces en los últimos 3 meses?)*
- Hotel ___ *(¿cuantas veces en los últimos 3 meses?)*
- Sauna ___ *(¿cuantas veces en los últimos 3 meses?)*
- Sala de cine para adultos ___ *(¿cuantas veces en los últimos 3 meses?)*
- Club sexual o Sala de videos ___ *(¿cuantas veces en los últimos 3 meses?)*
- Cabinas de Internet ___ *(¿cuantas veces en los últimos 3 meses?)*
- Peluquería ___ *(¿cuantas veces en los últimos 3 meses?)*
- Gimnasio o Campo atlético ___ *(¿cuantas veces en los últimos 3 meses?)*
- Baño publico ___ *(¿cuantas veces en los últimos 3 meses?)*
- Fiesta privada (en una casa/apartamento) ___ *(¿cuantas veces en los últimos 3 meses?)*
- Casa de travestis ___ *(¿cuantas veces en los últimos 3 meses?)*
- En mi casa ___ *(¿cuantas veces en los últimos 3 meses?)*
- En la casa de una pareja sexual ___ *(¿cuantas veces en los últimos 3 meses?)*
- Otro tipo de espacio publico o lugar social _______________ *(indique cual)* ___ *(¿cuantas veces en los últimos 3 meses?)*

10. En los últimos **<u>3 MESES</u>** ¿alguna vez tuvo sexo con más de una persona al mismo tiempo (por ejemplo una orgía u otro tipo de sexo grupal)?

(salte si la respuesta antes indica que no tuvo sexo en los últimos 3 meses)

a. No
b. Sí ___ *(¿cuantas veces en los últimos 3 meses?)*

AHORA, PIENSE EN LAS ULTIMAS <u>2 PERSONAS</u> CON QUIENES TUVO SEXO ALGUNA VEZ EN LOS ÚLTIMOS <u>3 MESES</u>.

LAS SIGUIENTES PREGUNTAS TRATAN DE LOS TIPOS DE LUGARES DONDE CONOCIÓ A ESTAS PAREJAS RECIENTES, DONDE TUVIERON SEXO, Y SUS ACTIVIDADES SEXUALES.

11. En los últimos **<u>3 MESES</u>**, ¿cuántas parejas sexuales tuvo en total?
Si no recuerda el número preciso, indique su mejor estimado.
    a. Ninguna (no tuve sexo desde hace más de 3 meses)
    b. Una
    c. Dos
    d. Tres (o más) ______ *(escriba cuantas)*

→ (*Según la respuesta, dirige el sujeto a responder al bloque completo del próximo ítem [#10] tantas veces como parejas indicadas acá, hasta el máximo de 2 veces)*

12. Pensando en su *<u>a. (última) / b. (penúltima)</u>* pareja sexual…
(Existe la posibilidad de responder 2 veces máximo al siguiente bloque de preguntas; depende del número de parejas que indicó en el último ítem)

Su *<u>a. (última) / b. (penúltima)</u>* pareja sexual fue un(a):
Marque sólo una opción
- Hombre
- Travesti, mujer transgénero o transexual
- Mujer (nacida mujer, no Trans)
- Hombre transgénero

¿Cuál es la mejor manera de describir a su *<u>a. (última) / b. (penúltima)</u>* pareja sexual?
Marque sólo una opción
- Pareja principal o estable
- Amigo cariñoso (o “amigo con derechos”)
- Pareja casual, con quien solo tuve sexo una vez
- Pareja casual, con quien tuve sexo más de una vez (o quizás tendré sexo otra vez en el futuro)
- Cliente (me pagó para tener sexo)
- Trabajador sexual (le pague para tener sexo)

Específicamente, ¿como **<u>conoció</u>** a su *<u>a. (última) / b. (penúltima)</u>* pareja sexual?
Recuerde que esta pregunta trata del lugar donde conoció a esta pareja sexual, pudiendo ser también el mismo lugar en donde tuvieron sexo o no.
Marque sólo una opción
- En línea (por ejemplo, páginas de internet o aplicaciones de citas)
- En uno de los lugares siguientes:
  - Disco / bar:
    - Sagitario Disco

    - La Cueva
    - Legendaris
    - Vale Todo Downtown
    - Perú Paris
    - La Agencia
    - 80 Divas
    - La Jarrita
    - Lola’s Bar
    - La Raffa
    - Amnesia
    - Kprichos
    - Andel Bar
    - Otra disco / bar _________ *(indique cual)*
- Espacio público afuera (plaza, calle, parque, centro comercial, playa):
    - Plaza San Martin
    - Parque Kennedy
    - Avenida Uruguay (cabinas)
    - Real Plaza Centro Cívico
    - Washington con Quilca
    - Playa _________ *(indique cual)*
    - Plaza Francia
    - Olivar de San Isidro
    - Parque Castilla
    - Boulevard de Miraflores
    - Otro/a plaza, calle, parque, centro comercial _________ *(indique cual)*
- Sauna:
    - Sauna 240
    - Sauna 69
    - Sauna Leonos
    - Sauna Olimpus
    - Sauna Sagitario
    - Sauna Zeus
    - Sauna Spartakus
    - Sauna Lobos
    - Sauna Esparta
    - Sauna Oupen
    - Otro sauna _________ *(indique cual)*
- Club Sexual o Sala de Videos:
    - Casanova
    - Inbox
    - Studio 5
    - Minotauro
    - Juguete
    - PK2
    - Taboo
    - Latinos
    - Otro club sexual o sala de videos _________ *(indique cual)*
- Cine Porno:
    - Cine Ritz
    - Cine Maximil

    - Otro cine porno _________ *(indique cual)*
- Cabina de Internet: _________ *(indique cual)*
- Peluquería: _________ *(indique cual)*
- Gimnasio o Campo atlético: _________ *(indique cual)*
- Baño público: _________ *(indique el barrio general)*
- Fiesta privada (en una casa/apartamento): _________ *(indique el barrio general)*
- Otro lugar específico _________ *(indique el barrio general)*

Específicamente, ¿dónde **tuvo sexo** con su _a. (última) / b. (penúltima)_ pareja sexual? *Recuerde que esta pregunta trata del lugar donde tuvo relaciones sexuales, pudiendo ser el mismo lugar en donde conoció a esta pareja inicialmente o un lugar diferente.*

Marque todas las opciones que apliquen

- Disco / bar:
    - Sagitario Disco
    - Vale Todo Downtown
    - 80 Divas
    - La Jarrita
    - Amnesia
    - Kprichos
    - Otro disco / bar _________ *(indique cual)*
- Sauna:
    - Sauna 240
    - Sauna 69
    - Sauna Leonos
    - Sauna Sagitario
    - Sauna Olimpus
    - Sauna Zeus
    - Sauna Spartakus
    - Sauna Lobos
    - Sauna Esparta
    - Sauna Oupen
    - Otro sauna _________ *(indique cual)*
- Club Sexual o Sala de Videos:
    - Casanova
    - Inbox
    - Minotauro
    - Studio 5
    - Juguete
    - PK2
    - Taboo
    - Latinos
    - Otro club sexual o sala de videos _________ *(indique cual)*
- Cine Porno:
    - Cine Ritz
    - Cine Maximil
    - Otro cine porno _________ *(indique cual)*
- Hotel:
    - Hotel Paraíso
    - Hotel Cisne
    - Hotel Andel

        - Q Hostel
        - Otro hotel _________ *(indique cual)*
    - Cabina de Internet: _________ *(indique cual)*
    - Peluquería: _________ *(indique cual)*
    - Gimnasio o Campo atlético: _________ *(indique cual)*
    - Baño publico: _________ *(indique el barrio general)*
    - Fiesta privada (en una casa/apartamento): _________ *(indique el barrio general)*
    - Casa de travestis: _________ *(indique el barrio general)*
    - Tuvimos sexo en mi casa
    - Tuvimos sexo en la casa de mi pareja
    - Otro lugar específico _________ *(indique el barrio general)*

Aproximadamente, ¿durante cuál parte del día estuvieron en el lugar donde tuvo sexo con su _*a. (última) / b. (penúltima)*_ pareja sexual? *Para esta pregunta y las siguientes, si tuvo sexo con esta pareja mas de una vez y en más de un solo lugar, marque su respuesta pensando solo en el primer lugar.*

Marque sólo una opción

a. Mañana (amanecer hasta mediodía)
b. Tarde (mediodía hasta la puesta del sol)
c. Noche (puesta del sol hasta medianoche)
d. Madrugada (medianoche hasta amanecer)
e. No recuerdo

¿Tuvo que pagar para entrar en este lugar donde tuvo sexo con su _*a. (última) / b. (penúltima)*_ pareja sexual?
(salta esta pregunta si antes respondió que tuvo sexo en su casa o la de su pareja)

a. No
b. Sí

De ser sí, ¿aproximadamente cuantos soles cobraron por ingresar?

- S 0-10
- S 10-20
- S 20-30
- S 30-40
- S 40-50
- Mas de S 50

En el lugar donde tuvo sexo con su _*a. (última) / b. (penúltima)*_ pareja sexual, ¿se proporcionaron condones?
(salta esta pregunta si antes respondió que tuvo sexo en su casa o la de su pareja)

a. No
b. Sí, hay condones en venta
c. Sí, proporcionaron condones gratis
d. No recuerdo

De ser no, Si se vendieran condones, ¿los compraría la próxima vez que vaya a este tipo de lugar en el futuro?

- Definitivamente sí
- Probablemente sí
- Quizás / No estoy seguro
- Probablemente no

- Definitivamente no

¿Usaría un condón si se los proporcionaran gratis en este lugar?

- Definitivamente sí
- Probablemente sí
- Quizás / No estoy seguro
- Probablemente no
- Definitivamente no

En el lugar donde tuvo sexo con su _*a. (última) / b. (penúltima)*_ pareja sexual, ¿se proporcionaron lubricantes?
(salta esta pregunta si antes respondió que tuvo sexo en su casa o la de su pareja)

a. No
b. Sí, hay lubricantes en venta
c. Sí, proporcionaron lubricantes a gratis
d. No recuerdo

De ser no, Si se vendieran lubricantes, ¿los compraría la próxima vez que vaya a este tipo de lugar en el futuro?

- Definitivamente sí
- Probablemente sí
- Quizás / No estoy seguro
- Probablemente no
- Definitivamente no

¿Usaría lubricantes si los proporcionaran gratis en este lugar?

- Definitivamente sí
- Probablemente sí
- Quizás / No estoy seguro
- Probablemente no
- Definitivamente no

Si le hubieran ofrecido la prueba para el VIH en el lugar donde tuvo sexo con su _*a. (última) / b. (penúltima)*_ pareja sexual, ¿lo habría considerado cuando estaba allá?
(salta esta pregunta si antes respondió que tuvo sexo en su casa o la de su pareja)

- Definitivamente sí
- Probablemente sí
- Quizás / No estoy seguro
- Probablemente no
- Definitivamente no

Si le hubieran ofrecido pruebas para otros tipos de enfermedades trasmitidas sexualmente (diferente de VIH), ¿lo habría considerado cuando estaba allá?

- Definitivamente sí
- Probablemente sí
- Quizás / No estoy seguro
- Probablemente no
- Definitivamente no

En el lugar donde tuvo sexo con su_*a. (última) / b. (penúltima)*_ pareja sexual, ¿se proporcionó alcohol o algún tipo de drogas?

(salta esta pregunta si antes respondió que tuvo sexo en su casa o la de su pareja)

a. No, no se proporcionó ni alcohol ni drogas
b. Sí, había alcohol
c. Sí, habían drogas _________ *(indique cuales)*
d. Sí, había alcohol y también drogas _________ *(indique cuales)*
e. No recuerdo

En el lugar donde tuvo sexo con su _*a. (última) / b. (penúltima)*_ pareja sexual, ¿habían cuartos privados (u otro sitio privado)?
(salta esta pregunta si antes respondió que tuvo sexo en su casa o la de su pareja)

a. Habían cuartos privados para tener sexo: es posible cerrar la puerta y también bloquearla.
b. Habían cuartos privados para tener sexo: es posible cerrar la puerta, pero no es posible bloquearla.
c. Habían cuartos semi-privados para tener sexo: hay una puerta pero no se puede cerrar completamente
d. Habían cuartos semi-privados para tener sexo: pero sin puerta
e. No habían cuartos individuales para tener sexo, sino que el sitio donde se puede tener sexo estaba en frente de otras personas
f. No recuerdo

¿En cuáles formas tuvo relaciones sexuales con su _*a. (última) / b. (penúltima)*_ pareja sexual?

Marque todas las opciones que apliquen e indique si usó condón o no en cada caso

- Sexo oral receptivo (usted se la chupó a él/ella) ____ (*de ser sí, ¿con condón?*)
- Sexo oral insertivo (él/ella se la chupó a usted) ____ (*de ser sí, ¿con condón?*)
- Sexo anal receptivo, como pasivo (él/ella se la metió por el ano a usted) ____ (*¿de ser sí, ¿con condón?*)
- Sexo anal insertivo, como activo (usted se la metió por el ano a él/ella) ____ (*de ser sí, ¿con condón?*)
- Sexo vaginal ____ (*¿de ser sí, ¿con condón?*)
- Masturbación (sin penetración) ____
- Otra forma _________ *(indique cual)* ____ (*¿de ser sí, ¿con condón?*)
- No recuerdo

Cuando tuvo sexo con su _*a. (última) / b. (penúltima)*_ pareja sexual, ¿fue con más de una sola persona (por ejemplo, una orgía u otro tipo de sexo grupal)?

a. No, fue solamente con una pareja al mismo tiempo
b. Sí, hubo sexo grupal y tuve sexo con penetración con más de una persona
c. Sí, hubo sexo grupal, pero solo tuve sexo con penetración con una sola pareja

LAS SIGUIENTES PREGUNTAS TRATAN DE SUS ACTIVIDADES SEXUALES MAS GENERALMENTE, Y TAMBIÉN TRATAN DEL VIH.

13. En los últimos **3 MESES**, ¿alguna vez tomó alcohol o utilizó alguna droga antes de o durante las relaciones sexuales?
    a. No
    b. Sí, tomé alcohol/utilicé drogas

Marque todas las opciones que apliquen

- Alcohol
- Marijuana
- Cocaína
- Anfetaminas
- Poppers
- Écstasis
- Heroína
- Ketamina
- Otra droga _________ *(indique cual)*

14. Antes de hoy, ¿Alguna vez se ha sometido a una prueba para detectar VIH?

    Marque sólo una opción

    a. No, nunca me he sometido a una prueba para detectar VIH
    b. Sí, y la última vez que me he sometido a esta prueba el resultado salió negativo (no tengo infección por VIH)
    c. Sí, y el resultado salió positivo (tengo infección por VIH)
       → *De ser sí*, ¿Está tomando el tratamiento antirretroviral?
       - No
       - Sí
    d. Sí, pero no recogí mi resultado (No estoy seguro si salió positivo o negativo)
    e. Prefiero no responder esta pregunta

Antes de hoy, ¿Cuándo fue la última vez en que se sometió a una prueba para detectar VIH?
Si ha tenido una prueba para el VIH hoy día, esta pregunta se refiere a la última vez ***antes de hoy****. Si no recuerda exactamente hace cuanto tiempo, indique su mejor estimado.*
(salta esta pregunta si respondió "a" o "e" a la última pregunta)

a. Dentro de los últimos 3 meses
b. Hace 3-6 meses
c. Hace 6-9 meses
d. Hace 9 meses a 1 año
e. Hace más de un año
f. Prefiero no responder esta pregunta

15. En los últimos **3 MESES**, ¿alguien **le ha pagado a usted** por tener sexo?

    a. No
    b. Sí

16. En los últimos **3 MESES**, ¿**usted le ha pagado a** alguien por sexo?

    a. No
    b. Sí

17. ¿Se considera trabajador/a sexual?

    a. No
    b. Sí

FINALMENTE, LAS SIGUIENTES PREGUNTAS TRATAN DE SUS CONOCIMIENTOS ACERCA DE LA PREVENCION DEL VIH.

18. ¿Alguna vez ha oído de Profilaxis de Pre-Exposición (“PrEP”) para el VIH?
    a. No
    b. Sí

19. ¿Piensa que la siguiente frase es verdadera o falsa?:
    "Es muy improbable que una persona con VIH transmita el virus a su pareja sexual si es que la persona con VIH está tomando tratamiento antirretroviral y ha logrado que el virus no se detecte en su sangre"
    Si no se siente seguro de la respuesta correcta, responda “No sé”
    a. Verdadera
    b. Falsa
    c. No sé
